# Zika virus infection perturbs osteoblast function

**DOI:** 10.1038/s41598-018-35422-3

**Published:** 2018-11-19

**Authors:** Noreen Mumtaz, Marijke Koedam, Petra B. van den Doel, Johannes P. T. M. van Leeuwen, Marion P. G. Koopmans, Bram C. J. van der Eerden, Barry Rockx

**Affiliations:** 1 Department of Viroscience, Erasmus University Medical Centre, Rotterdam, The Netherlands; 2 Department of Internal Medicine, Erasmus University Medical Centre, Rotterdam, The Netherlands

## Abstract

Zika virus (ZIKV) infection is typically characterized by a mild self-limiting disease presenting with fever, rash, myalgia and arthralgia and severe fetal complications during pregnancy such as microcephaly, subcortical calcifications and arthrogyropsis. Virus-induced arthralgia due to perturbed osteoblast function has been described for other arboviruses. In case of ZIKV infection, the role of osteoblasts in ZIKV pathogenesis and bone related pathology remains unknown. Here, we study the effect of ZIKV infection on osteoblast differentiation, maturation and function by quantifying activity and gene expression of key biomarkers, using human bone marrow-derived mesenchymal stromal cells (MSCs, osteoblast precursors). MSCs were induced to differentiate into osteoblasts and we found that osteoblasts were highly susceptible to ZIKV infection. While infection did not cause a cytopathic effect, a significant reduction of key osteogenic markers such as *ALP*, *RUNX2*, calcium contents and increased expression of IL6 in ZIKV-infected MSCs implicated a delay in osteoblast development and maturation, as compared to uninfected controls. In conclusion, we have developed and characterized a new *in vitro* model to study the role of bone development in ZIKV pathogenesis, which will help to identify possible new targets for developing therapeutic and preventive measures.

## Introduction

Zika virus (ZIKV) is an arthropod borne (arbo) virus and belongs to the family of *Flaviviridae*. Since its discovery in Uganda in 1947, ZIKV has invaded different territories around the world. In 2015, ZIKV was reported in Brazil resulting in an epidemic of unprecedented scale in the Americas and the Caribbean^1,2^. Severe complications due to ZIKV infection are observed during pregnancy, including congenital abnormalities such as microcephaly and subcortical calcifications^3^. In adults, acute symptomatic ZIKV infection is typically characterized by a self-limiting disease with fever, rash, conjunctivitis, myalgia and arthralgia/arthritis^4^. Arthralgia is reported in over 70% of symptomatic ZIKV cases including persistent or recurrent arthralgia for more than 30 days^5,6^.Virus-induced arthralgia has previously been reported following infection with the alphaviruses Ross River virus (RRV) and chikungunya virus (CHIKV)^7,8^. Infection with RRV can perturb osteoblast function and trigger pathologic bone loss due to induction of interleukin-6 (IL6), and contributes to virus-induced arthritis^7^. Osteoblasts are responsible for the deposition of bone matrix and mineralization of the bone matrix. However, osteoblast function may be altered due to infection as reported previously for other viruses such as hepatitis C virus (HCV), measles virus (MV) and human immunodeficiency virus (HIV)^9^.

ZIKV has been detected in synovial fluid of a patient with arthralgia^10^, and a recent study has shown the susceptibility of an osteoblast-like cell line for ZIKV infection^11^. Osteoblasts originate from mesenchymal stem cells (MSCs) and in a recent case of miscarriage associated with ZIKV infection, ZIKV displayed tropism for fetal MSC in the perichondrium^12^. However, it is not known if MSC- derived osteoblasts are susceptible to ZIKV infection, and whether infection of osteoblasts affects their function thereby contributing to ZIKV pathogenesis and ZIKV-associated osteoarticular complications. Therefore, in the current study, we determined the susceptibility of primary human MSC-derived osteoblasts to infection with ZIKV and its effect on differentiation, maturation and function of these cells.

## Results

### ZIKV infects osteoblasts with high titers

**Figure 1:**
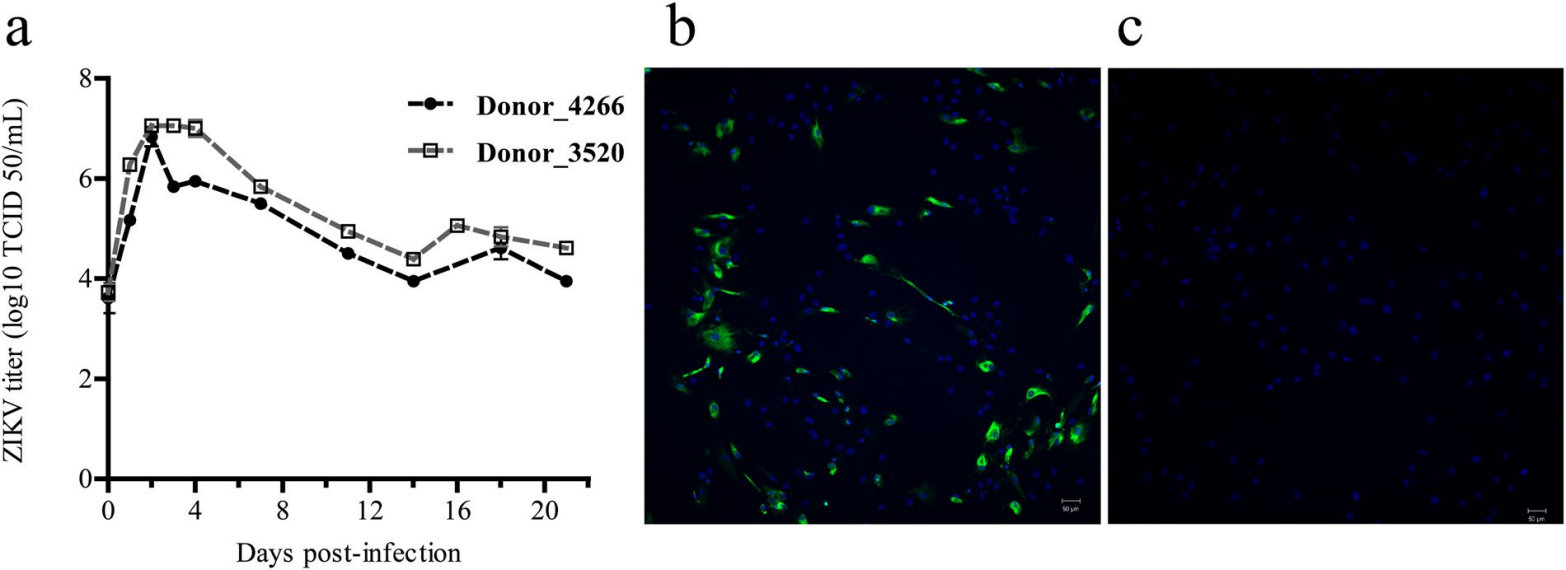
Replication of ZIKV in primary osteoblasts. Culture supernatant was collected at different time points after infection of primary osteoblasts by ZIKV (moi = 5). (**a)** Growth curve kinetics of ZIKV infection in osteoblasts from Donor 4266 (closed circles) and Donor 3520 (open squares) during differentiation over the period of 3 weeks. Error bars represent the standard error of mean (S.E.M). (**b)** Representative immunofluorescent images of ZIKV-infected cells stained for ZIKV antigen (green) and nuclei (blue), and (**c)** uninfected controls at day 4 post-infection. Magnification 200x.

In order to determine whether the osteoblasts are susceptible to infection with ZIKV, we infected osteoblast cultures with ZIKV at an moi of 5. The infected cultures did not show evidence for a cytopathic effect (CPE) due to ZIKV infection throughout the culture period (Supplementary Fig. 1). CHIKV infection resulted in extensive CPE within 4 days post-infection and as such samples were not available for subsequent analyses. Similar to ZIKV, DENV infection did not result in CPE (supplementary Fig. 4). ZIKV infection of osteoblasts was confirmed by IFA at day 4, 7, 11 and 17 post-infection (Fig. 1 and Supplementary Fig. 2). Interestingly, despite using a high moi, not all cells were infected with ZIKV and the level of ZIKV infection remained similar throughout the experiment. However, ZIKV infected differentiating osteoblasts and produced high infectious titers of up to 10^7^ TCID_50_/ml within 2 days post infection. No significant evidence of DENV infection was observed by IFA or virus titration (Supplementary Figs 3–4). Virus growth kinetics and IFA during the differentiation period showed that osteoblast cultures were persistently infected with ZIKV and shedding of infectious virions was observed over the period of three weeks post-infection for both donors (Fig. 1a–c).

### ZIKV perturbs osteoblast differentiation and mineralization

**Figure 2:**
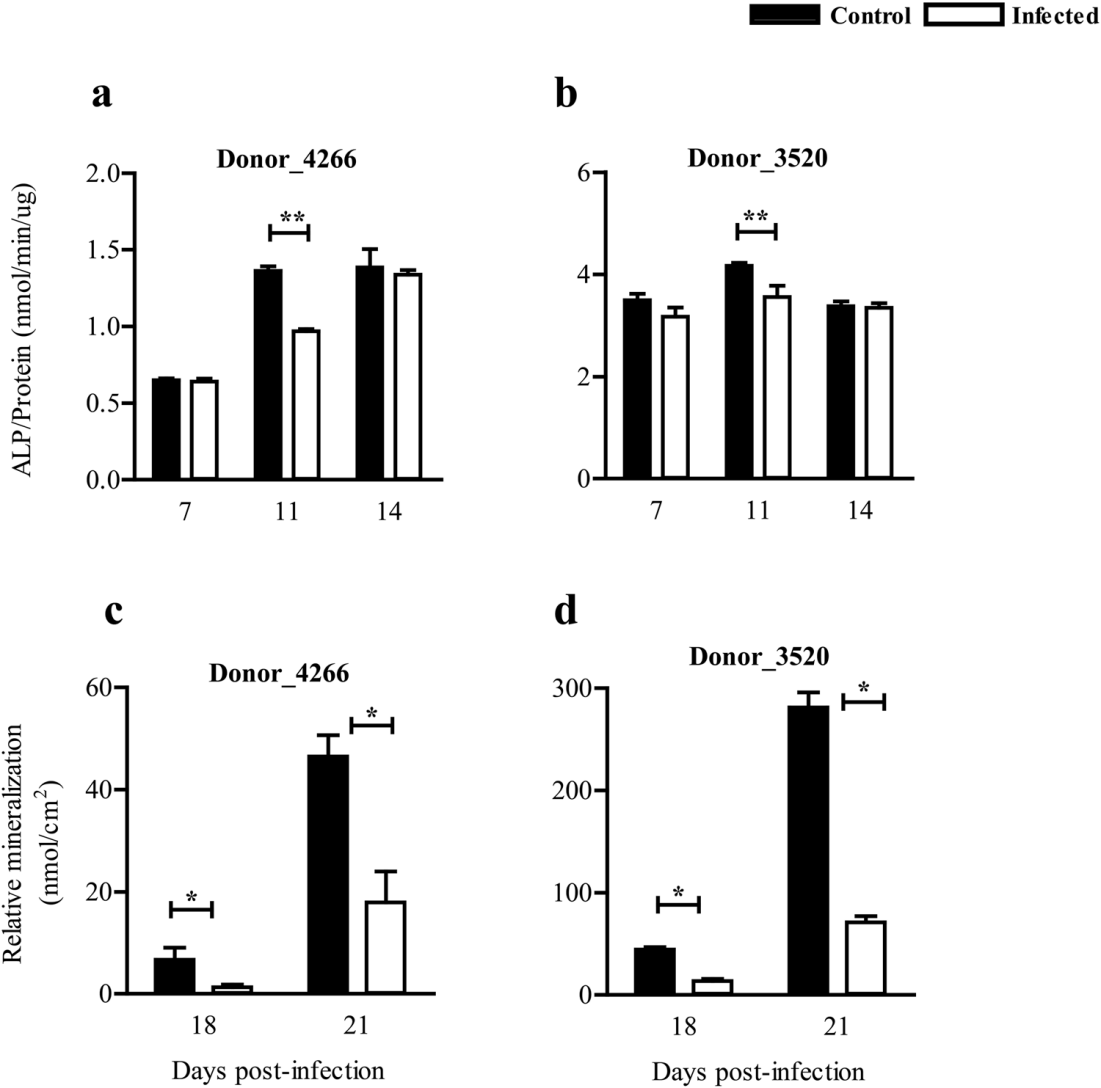
Effect of ZIKV infection on osteoblast differentiation and maturation. Effect of ZIKV infection on osteoblast differentiation is measured by alkaline phosphatase (ALP) activity in cultures from (**a)** Donor 4266 and (**b)** Donor 3520, and effect on mineralization is measured as a concentration of calcium present in the cultures from **c)** Donor 4266 and (**d)** Donor 3520. Results are compared between ZIKV -infected (white bars) and uninfected controls (black bars) and ALP levels are normalized against total protein. Error bars represent the standard error of mean. *p < 0.05.

The effect of ZIKV infection on the differentiation and maturation of osteoblasts was determined in infected cultures by quantifying the ALP activity and mineral content at different time points (Day 7, 11, 14, 18 and 21) post infection. These time points were selected as optimal time points for quantification of differentiation and maturation of MSC-derived osteoblasts, based on previous studies^13,14^. In ZIKV-infected osteoblasts derived from 2 different MSC donors, ALP activity was significantly reduced at day 11 post-infection compared to uninfected controls (Fig. 2a,b). Additionally, ZIKV infection significantly reduced osteoblast maturation in terms of mineral content in ZIKV-infected osteoblasts compared to uninfected controls at day 18 and 21 post-infection (Fig. 2c,d).

### ZIKV affects osteoblast marker genes

**Figure 3:**
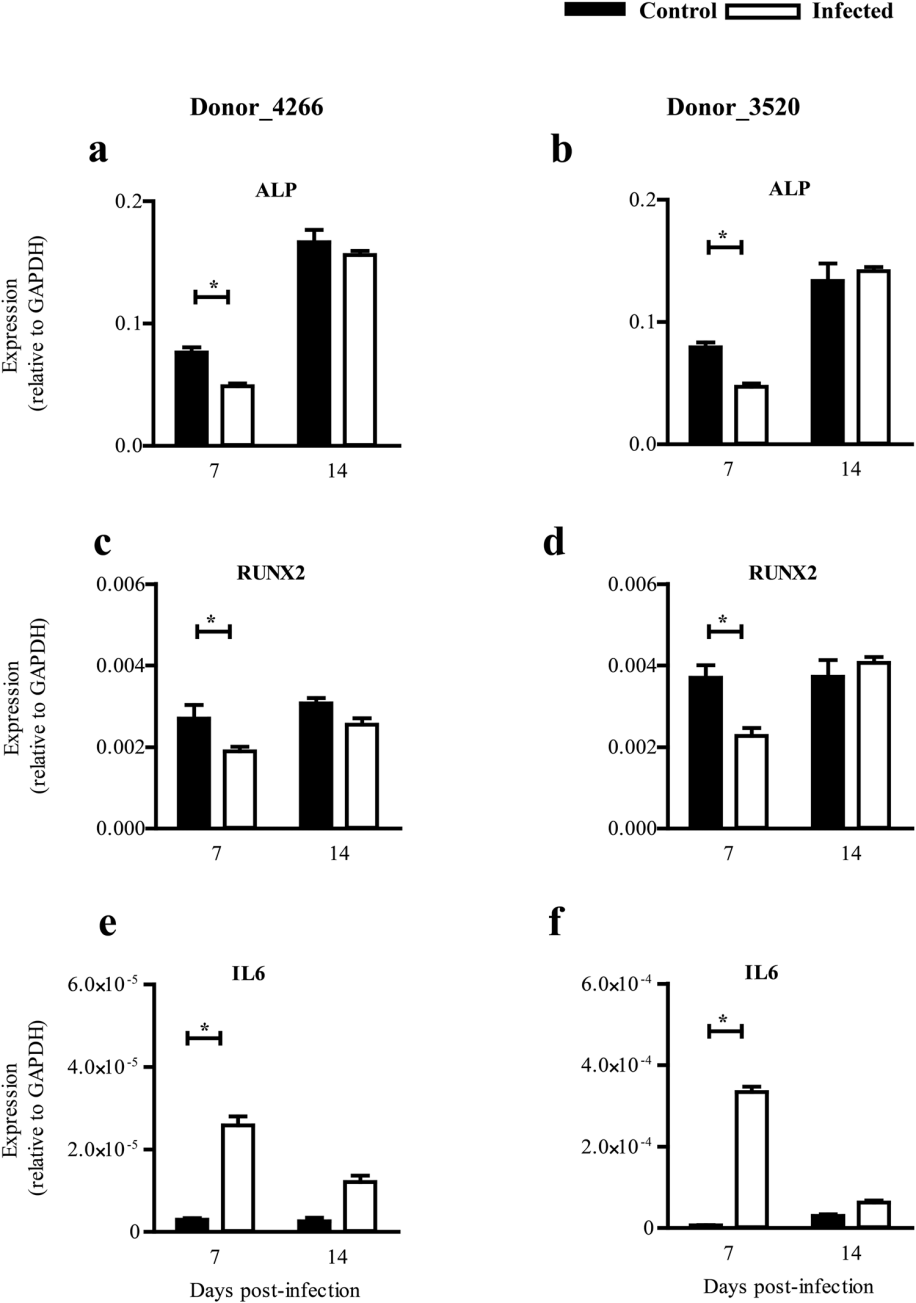
Gene expression levels of analysed genes after ZIKV infection. Gene expression of key transcription factors from ZIKV infected osteoblasts (white bars) versus uninfected controls (black bars) in (Left panel) Donor 4266 and (Right panel) Donor 3520. Gene expression was corrected for house keeping gene, *GAPDH*. Error bars represent the standard error of mean. *p < 0.05.

To quantify the impact of ZIKV infection on the expression levels of key factors during ZIKV infection, which play an instrumental role in determining the osteoblasts phenotype, we quantified gene expression of *ALP*, Runt-related transcription factor 2 (*RUNX2*, a key transcription factor for osteoblast differentiation) and a classical inflammatory mediator interleukin 6 (*IL6*). A significant reduction of *ALP* (Fig. 3a,b) and *RUNX2* expression (Fig. 3c,d) was observed in ZIKV-infected differentiating osteoblasts compared to uninfected controls (p value < 0.05) at day 7 post-infection. Interestingly, the levels of *IL6* were significantly increased in infected osteoblasts (Fig. 3e,f).

## Discussion

The development of arthralgia/arthritis has been a hallmark clinical symptom in several arbovirus infections, including ZIKV, CHIKV and dengue viruses^9,15,16^. While generally not considered as severe as in CHIKV infection, arthralgia/arthritis has been reported in over 70% of ZIKV cases^6^. In this study we investigated the susceptibility of MSC-derived osteoblasts to ZIKV infection and the effects of this infection on the phenotype of osteoblasts.

ZIKV readily infects differentiating primary human osteoblasts and grows to high titers within 2 days post infection. These findings are in line with a recent report which confirms the susceptibility of a human osteoblast-like cell line (HOBIT) to ZIKV^11^, as well as observations from other arthritogenic arboviruses such as CHIKV^17^. However, in contrast to previous studies we did not observe CPE in ZIKV-infected primary osteoblasts. This disparity may occur in part due to the differences in *in vitro* models, as primary osteoblasts derived from MSCs were used in the current study compared to an osteoblast-like cell line in the previous ZIKV study and bone fragment-derived osteoblasts used in the CHIKV study. Additionally, flavivirus replication has been shown to incompletely inhibit host cell macromolecular synthesis, which may result in non-cytopathic persistent infections^18–20^. In the absence of CPE, ZIKV infection resulted in a persistent infection of osteoblasts for up to 3 weeks post infection, which suggests there may be viral persistence similar to CHIKV^17^.

Osteoblasts play an important role in bone remodeling, which is a tightly regulated process, requiring a balance in bone resorption and bone formation. Interestingly, persistent ZIKV infection had a direct effect on the differentiation, maturation and function of primary osteoblasts. Osteoblast differentiation comprises a highly coordinated sequence of events and is regulated by the activities of several key transcription factors. The key transcription regulator RUNX2 plays a central role by modulating the commitment of osteoprogenitors and expression of major bone matrix genes by activating different pathways^21^. Following osteoblast commitment, increased levels of ALP are considered as the marker of osteogenic differentiation as it is one of the first functional enzymes observed prior to the process of mineralization. Indeed, by reducing levels of the mineralization inhibitor pyrophosphate, ALP activity is critical for the mineralization process^22^. Thus, our findings showing significantly reduced expressions of RUNX2 and ALP in persistently infected osteoblasts followed by diminished mineral depositions are of particular interest in view of ZIKV-induced alterations in the osteoblast phenotype.

In addition to bone formation biomarkers, elevated levels of IL6 comparable to that described for other arthritogenic arboviruses were also observed following ZIKV infection. This key pro-inflammatory mediator plays a pivotal role in the pathophysiology of rheumatoid arthritis, where it has been associated with stimulating neutrophil migration, osteoclast maturation and pannus proliferation^23^. During alphavirus infection, IL6 has been shown to indirectly disturb the bone homeostasis by stimulating the induction of bone resorption mediators in osteoblast cultures, resulting in bone loss and joint inflammation^7,17^. Future studies will focus on identifying key biomarkers which will allow us to predict the osteoarticular complications and disease severity following ZIKV infection.

In conclusion, these data clearly demonstrate that osteoblasts are susceptible to infection by ZIKV and that infection results in reduced differentiation and maturation of osteoblasts. The impaired function of osteoblasts can subsequently trigger an imbalance in bone homeostasis and induce bone-related disorders. These data warrant further studies to delineate the molecular mechanisms behind ZIKV pathogenesis in bone development *in vitro* and *in vivo*.

## Materials and Methods

### Cell cultures

Human bone marrow-derived MSCs of two healthy donors (Donor 4266 and 3520, 33 and 20 year old males, respectively) were purchased from Lonza (PT-2501). MSCs were differentiated into osteoblasts as described previously^24^. Briefly, MSCs were cultured in αMEM medium (Gibco, Thermofisher) supplemented with 10% heat-inactivated fetal calf serum a (FCS, Sigma), 20 mM Hepes (Sigma), 100 U/mL penicillin (Lonza) and 100 µg/mL streptomycin (Thermofisher) and 1.8 mM CaCl_2_ (Sigma) at 37 °C and 5% CO_2_ in a humidified atmosphere. Medium on day 3 post-seeding was supplemented with 100 mM dexamethasone (dex) and 10 mM β-glycerophosphate (osteogenic medium) resulting in differentiation into mineralizing osteoblasts within 2–3 weeks^14^. The media were refreshed twice per week. Vero cells (African green monkey kidney epithelial cells, ATCC CCL-81) were cultured in Dulbecco’s modified Eagle’s medium (DMEM, Lonza, the Netherlands) supplemented with 10% heat-inactivated fetal bovine serum (FBS, Greiner Bio-One, Austria), 2 mM L-glutamin (Lonza), 1% sodium bicarbonate (Lonza), 1% Hepes (Lonza), 100 U/mL penicillin (Lonza) and 100 µg/mL streptomycin (Lonza) at 37 °C and 5% CO_2_ in a humidified atmosphere.

### Virus

Zika virus Suriname ZIKVNL00013 (ZIKVAS-Sur16) was isolated from a patient in The Netherlands (EVAg no. 011V-01621)^12^. This strain was previously shown to have a similar phenotype as the prototypic Asian lineage of ZIKV^25^. The virus stock used in this study was grown in Vero cells and passage number 3 was used for the current study. Virus titers in the supernatants were determined by endpoint titrations on Vero cells as described previously^25^. In some experiments, dengue virus (DEN 2 16681 strain) and chikungunya virus (CHIKV/IND/NL10/152) were included as controls. Briefly, tenfold serial dilution were inoculated onto a monolayer of Vero cells in a 96-wells plate (2 × 10^4^ cells/well). Cytopathic effect (CPE) was used as read out and determined at 5 days post-infection (dpi), and virus titers were calculated as the 50% tissue culture infective dose (TCID50) using the Spearman-Kärber method^26^. An initial 1:10 dilution of supernatant resulted in a detection limit of 10^1.5^ TCID50/ ml.

### Replication kinetics of ZIKV

MSCs were seeded three days prior to infection (day = −3). Three days post-seeding (day = 0), MSCs were stimulated into osteoblasts by adding osteogenic medium. After 6 hours of stimulation, osteoblast cultures were infected at a multiplicity of infection (moi) of 5 with ZIKVfor 1 hour at 37 °C in 5% CO_2_. The moi was based on titers determined on Vero cells. After incubation, the supernatant was removed and cells were washed three times with αMEM medium containing 10% heat-inactivated FCS followed by osteogenic medium and cells were cultured twice weekly up to three weeks depending on the experiment. Uninfected controls were cultured in parallel. To determine the ZIKV infectious titers produced, cell supernatants were collected at different time points post infection. Supernatant was stored at −80 °C until further use. Experiments were performed in triplicate with two different MSC donors.

### Immunofluorescence assay

Infected cells from the replication growth kinetics assay were fixed with 4% PFA at days 4, 7, 11 and 17 post infection, permeabilized with 70% ethanol and stained using an immunofluorescence assay (IFA) as described previously^25^. Briefly, cells were incubated with mouse monoclonal antibody anti-flavivirus group antigen (MAB10216) clone D1–4G2-4-15 (Millipore, Germany) followed by staining with goat anti-mouse IgG conjugated with Alexa Fluor 488 (Life technologies, the Netherlands). After incubation, cells were mounted in ProLong® Diamond Antifade Mountant with DAPI (Life technologies, USA). Uninfected cells and ZIKV-infected cells stained with mouse isotype IgG2a antibody (Dako, Denmark) were used as negative controls. ZIKV-infected cells were identified by using a Zeiss LSM 700 confocal laser scanning microscope fitted on an Axio observer Z1 inverted microscope (Zeiss, Breda, the Netherlands). All images were processed using Zen 2010 software (Zeiss).

### Alkaline phosphatase, mineralization, and protein assays

Alkaline phosphatase (ALP) and calcium measurements were performed at different time points based on gene expression data as described previously^13,14^. Briefly, ALP activity was determined by an enzymatic reaction, where the ALP-mediated conversion of para-nitrophenylphosphate (pNPP) (Sigma) to paranitrophenol (PNP) during 10 min at 37 °C was measured at 405 nm. ALP results were adjusted for protein content of the cell lysates. For protein measurement, 200 µL of working reagent was added to 25 µL of cell lysate. The mixture was incubated for 30 min at 37 °C, cooled down to room temperature (RT) for 10 min, and absorbance was measured at 595 nm. For calcium measurements, cell lysates were incubated overnight with 0.24 M HCl at 4 °C. Calcium content was determined colorimetrically using a calcium assay reagent prepared by combining 1 M ethanolamine buffer (pH 10.6) with 0.35 mM O-cresolphthalein complex one in a ratio of 1:1. All measurements were performed using a Victor2 plate reader.

### Quantification of mRNA expression

RNA was extracted from ZIKV-infected and uninfected cells at different time points during osteoblast differentiation using TRIzol reagent (Thermo Fisher Scientific). RNA isolation, cDNA synthesis, and PCR reactions were performed as described previously^24^. Oligonucleotide primer pairs were designed to be either on exon boundaries or spanning at least one intron (Table 1). Gene expression was corrected for the housekeeping gene glyceraldehyde 3-phosphate dehydrogenase (GAPDH).

**Table 1:** Primer sequences of the analyzed genes.

Gene	Forward primer (5′-3′)	Reverse primer (5′-3′)
GAPDH	CCGCATCTTCTTTTGCGTCG	CCCAATACGACCAAATCCGTTG
ALP	TAAAGCAGGTCTTGGGGTGC	GGGTCTTTCTCTTTCTCTGGCA
RUNX2	GATTACAGACCCCAGGCAGG	GGCTCAGGTAGGAGGGGTAA
IL6	AAAGAGGCACTGGCAGAAAA	TTTCACCAGGCAAGTCTCCT

GAPDH, Glyceraldehyde 3-phosphate dehydrogenase; ALP, Alkaline phosphate; RUX2, Runt- related transcription factor 2; IL6, Interleukin 6.

### Statistical analysis

The statistical analyses were performed using GraphPad Prism 5.01 software. All results are expressed as means with standard error of the mean (S.E.M.). Mann Whitney U test was used for the comparison between two groups (infected versus un-infected). *P* value ≤ 0.05 was considered significant.

## Electronic supplementary material

suppelmentary file

## Data Availability

The data sets generated during and/or analyzed during the current study are available with the corresponding author, and can be accessed on reasonable request.
