# Supplementary material for: Zika virus infection perturbs osteoblast function
Source: Sci Rep. 2018 Nov 19;8:16975. doi: 10.1038/s41598-018-35422-3 (PMC6242880; doi:10.1038/s41598-018-35422-3)
Supplement: Supplementary file 1 — suppelmentary file [file 41598_2018_35422_MOESM1_ESM.docx]

**Title: Zika virus infection perturbs osteoblast function**

**Authors list:**

Noreen Mumtaz^1^, Marijke S. Koedam^2^, Petra B. van den Doel^1^, Johannes P.T.M. van Leeuwen^2^, [Marion P. G. Koopmans](https://www.ncbi.nlm.nih.gov/pubmed/?term=Koopmans%20MP%5BAuthor%5D&cauthor=true&cauthor_uid=28815211)^1^ Bram C.J. van der Eerden *^2^ and Barry Rockx *^1^

Authors affiliation:

^1^Department of Viroscience, Erasmus University Medical Centre, Rotterdam, the Netherlands

^2^Department of Internal Medicine, Erasmus University Medical Centre, Rotterdam, the Netherlands

*Authors contributed equally

Corresponding authors

Barry Rockx

Department of Viroscience

Erasmus MC, Rotterdam

Email: b.rockx@erasmusmc.nl

P.O.Box 2040, 3000 CA Rotterdam, The Netherlands, internal postal address Ee1722.

Telephone +31 10 704 40 68

Bram van der Eerden

Department of Internal Medicine

Erasmus MC, Rotterdam

Email: b.vandereerden@erasmusmc.nl

P.O.Box 2040, 3000 CA Rotterdam, The Netherlands, internal postal address Ee585b.

Telephone +31 10 7032841

**
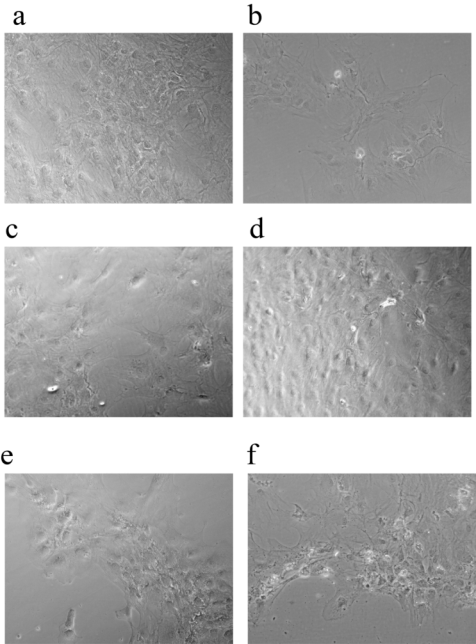
**

**Supplementary figure 1:** ZIKV infection in differentiating osteoblasts without CPE. Representative bright field images of non-infected controls and ZIKV infected osteoblasts at day 7 **(a, b)**, 11 **(c, d)** and 17 **(e, f)** post infection. Magnification 200x.

**
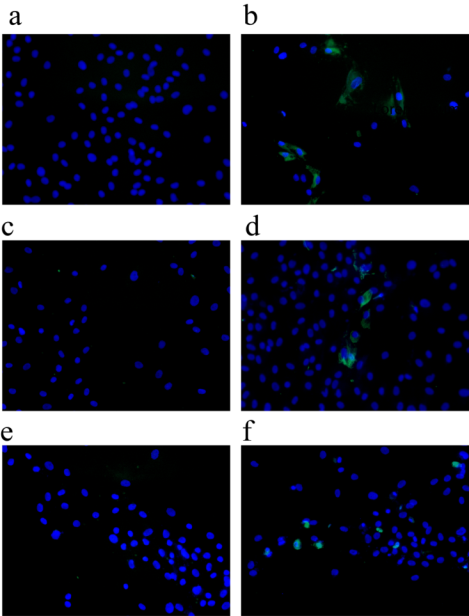
**

**Supplementary figure 2:** Persistent ZIKV infection in differentiating osteoblasts. Representative immunofluorescent images of osteoblasts stained for ZIKV antigen (green) and nuclei (blue). Non-infected osteoblast control and ZIKV-infected osteoblasts at day 7 **(a, b)**, 11 **(c, d)** and 17 **(e, f)** post infection**.** Magnification 200x.


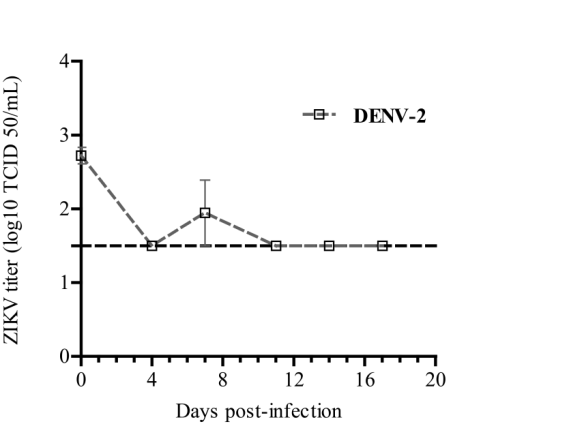


**Supplementary figure 3:** DENV 2 infection of osetoblasts. Culture supernatant was collected at different time points after infection of primary osteoblasts by DENV 2 (moi= 5). Growth curve kinetics of DENV 2 infection in osteoblasts from Donor 3520 (open squares) during differentiation over the period of 3 weeks. Errors bar represent the standard error of mean (S.E.M ).


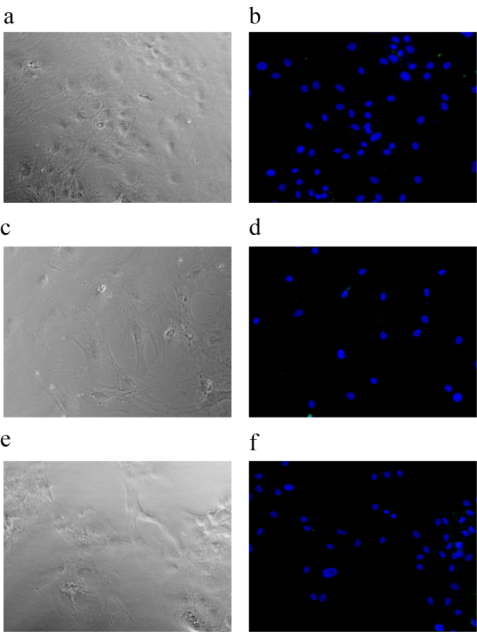


**Supplementary figure 4:** . Representative bright field and immunofluorescent images of osteoblasts stained for DENV 2 antigen (green) and nuclei (blue). DENV 2 -infected osteoblasts at day 7 **(a-b)**, day 11 (**c-d)** and day-17 **(e-f)** post infection**.**  Magnification 200x.
